# Needle-free, Novel Fossa Ovalis Puncture with Percutaneous Transluminal Coronary Angioplasty Guidewire and Microcatheter in Pigs and a Human with an Extremely Tortuous Inferior Vena Cava

**DOI:** 10.31083/j.rcm2505170

**Published:** 2024-05-14

**Authors:** Guang-Xia Wang, Hong Luo, Feng-Peng Jia, Run-Tu Li, Quan He, Chun-Chang Qin

**Affiliations:** ^1^Department of Cardiology, The First Affiliated Hospital of Chongqing Medical University, 400017 Chongqing, China

**Keywords:** fossa ovalis, microcatheter, percutaneous transluminal coronary angioplasty guidewire, needle-free, transseptal puncture

## Abstract

**Background:**

Transseptal puncture (TSP) performed with the Brockenbrough (BRK) needle is technically demanding and carries potential risks. The back end of the percutaneous transluminal coronary angioplasty (PTCA) guidewire is blunt and flexible, with good support, it can puncture the right ventricle-free wall, which is thicker than the atrial-septum. The guidewire is thin and easy to manipulate. This study evaluated the performance of TSP with a PTCA guidewire and microcatheter without a needle.

**Methods:**

The back end of a PTCA guidewire was advanced into the Tiger (TIG) catheter, within the SL1 sheath, to puncture the fossa ovalis (FO) under fluoroscopy. Subsequently, the microcatheter was inserted into the left atrium (LA) above the guidewire, and the front end of the guidewire was exchanged in the LA. After the puncture site was confirmed by contrast, the TIG catheter and a 0.032 inch wire were advanced into the LA. Finally, the sheath, with the dilator, was advanced over the wire into the LA. The safety margin of this method was tested in a pig model.

**Results:**

The puncture was successful in all seven pigs tested with a puncture-to-sheath entry time of <20 minutes and no procedure-related complications. The method was successfully used to perform a difficult TSP in a patient with an extremely tortuous inferior vena cava, in whom puncture with a BRK needle had repeatedly failed.

**Conclusions:**

Cardiologists may use the PTCA guidewire and microcatheter as an alternative to the needle while performing TSP in special conditions, such as an extremely tortuous inferior vena cava.

## 1. Introduction

In recent years, the number of transseptal transcatheter interventions requiring transseptal puncture (TSP) has grown exponentially. More and more interventional cardiac procedures will be performed in less experienced centers and many new physicians will need to complete TSP procedures. The transseptal method has remained largely unchanged for decades and is based on the use of a rigid, metal Brockenbrough (BRK) needle [1, 2]. Although Ross [3] designed the puncture needle to directly measure left atrium (LA) pressure, the needle is not used for this purpose today. The long and rigid needle may puncture or tear the surrounding tissue and cause life-threatening complications due to its bulkiness and difficulty in operation. Complication rates with this needle vary between 0.0% and 6.7% [4].

Because TSP has widespread use in cardiac intervention procedures, various instruments have been created to help improve the safety and efficiency of the procedure, such as the SafeSept guidewire, the radiofrequency (RF) needle, transesophageal echocardiography (TEE), intracardiac echocardiography (ICE), and three-dimensional (3D) electroanatomic mapping [2, 5, 6]. Although progressive techniques are of great help for TSP, they do not guarantee its safety or success [7, 8], and some of the equipment is expensive and requires specialized personnel, which are not available at many hospitals.

TSP is technically demanding with the conventional puncture needle. Even in experienced hands, this may result in severe complications or inability to accomplish the procedure in complex cases. Many new inexperienced physicians will need to complete TSP procedures, therefore, any effort to reduce the risk is worthwhile.

Although the back end of a percutaneous transluminal coronary angioplasty (PTCA) guidewire is flexible and blunt, it can puncture through the thick right ventricle (RV)-free wall with the backup of a guiding catheter [9]. Because the guidewire is thin and flexible, it can be easily and safely manipulated. In this study, TSP was performed with the PTCA guidewire and microcatheter without a puncture needle. The method was successful in a pig model and in one patient presenting with an extremely tortuous inferior vena cava, who failed TSP with a BRK needle.

## 2. Materials and Methods

The experimental protocol for this study was approved by the Institutional Animal Care and Use Committee of the First Affiliated Hospital of Chongqing Medical University (ethics number: 2019-117). The care and use of the animals in this study strictly adhered to the guidelines outlined in the Guide for the Care and Use of Laboratory Animals. Furthermore, this research was approved by the Ethics Committee of the First Affiliated Hospital of Chongqing Medical University (ethics number: 2021-280), and it was conducted in accordance with the ethical standards laid down in the 1964 Declaration of Helsinki and its later amendments. Informed consent was obtained from the human participant involved in this study.

Animals were anesthetized using 0.04 mg/kg atropine and 0.6 mg/kg midazolam intramuscularly, and intravenous propofol (60–300 mg/h) was used to maintain anesthesia. A femoral artery sheath was used to measure arterial blood pressure. ${\mathrm{SiO}}_{2}$ levels of the tail and electrocardiograms were observed. The intervention was carried out with the aid of a fluroscope. All the pigs received heparin (60 U/kg intravenous) after the sheath was implanted.

### 2.1 The TSP Procedure

Step 1: Reaching the fossa ovalis (FO) landmark. Before TSP was performed, a steerable decapolar mapping catheter (APT Medical Co., Shenzhen, China) was inserted and placed into the coronary sinus (CS) through the left jugular vein as a TSP landmark. Next, an 8.5 Fr transseptal sheath (St. Jude Medical, Minneapolis, MN, USA) with its dilator was introduced and advanced from the right femoral vein to the superior vena cava over a 135 cm long, 0.032 inch “J” guidewire. Then the guidewire was removed; the sheath and dilator were withdrawn while rotating to point the sheath tip toward the 5 o’clock position under X-ray guidance until the two “jumps” of the tip were observed, identifying the location of the FO. In the left anterior oblique (LAO) 30° projection, the FO was at 1 pyramidal height above the ostia of the CS, with the pigs positioned in the left recumbent position. The dilator was withdrawn from the sheath. A 5 Fr Tiger (TIG) angiographic catheter (Terumo) was advanced into the SL1 sheath that reached the FO from the femoral vein over a standard “J” guidewire. Then the “J” guidewire was removed and the back end of the 0.014 inch Sion PTCA guidewire (Asahi Intec, Seto, Japan) within a 2.3 Fr Cosair microcatheter was advanced into the TIG catheter.

Step 2: TSP. The back end of a PTCA guidewire within a microcatheter was advanced into the TIG angiographic catheter to puncture the FO and enter the LA. Once the PTCA guidewire had passed the FO by several centimeters, the microcatheter was advanced over the wire into the LA, and a small amount of contrast was injected to localize the tip of the microcatheter in the LA.

Step 3: Obtain LA access. The soft front end of a PTCA guidewire was exchanged within the microcatheter, and the 5 Fr TIG catheter was advanced over the microcatheter (including the PTCA guidewire) into the LA. Once the TIG angiographic catheter crossed the FO, the microcatheter and guidewire were withdrawn, and a “J” guidewire was advanced into the left superior pulmonary vein (LSPV) area to guide the dilator and sheath into the LA. Finally, the sheath entered the LA, and the dilator and “J” guidewire were gently withdrawn. At this time, LA access was obtained.

### 2.2 Safety Experiment

To assess the safety of this method, the PTCA guidewire intentionally punctured the The left atrial appendage (LAA). The LAA was chosen because it is at high risk for bleeding after an injury. The puncture site was then expanded with the balloon (1.5 mm × 15 mm) (APT Medical Co., Shenzhen, China) for 15 minutes and then the balloon was withdrawn. The animals were closely monitored by echocardiography for any signs of pericardial effusion.

Data were analyzed using SPSS (v21.0, IBM, Armonk, NY, USA) and are reported as mean ± standard deviation unless otherwise indicated.

## 3. Results

Seven male Yorkshire swines (mean weight 40.4 ± 7.6 kg; range: 30–50 kg) subjected to TSP were included in the study as the animal models. In all cases, the puncture was successful with no complications. Subsequently, we encountered a patient with an extremely tortuous inferior vena cava that was difficult for the BRK needle to reach the FO. The TSP was successful performed with this method.

### 3.1 Animal Experiment

**Fig. 1.**
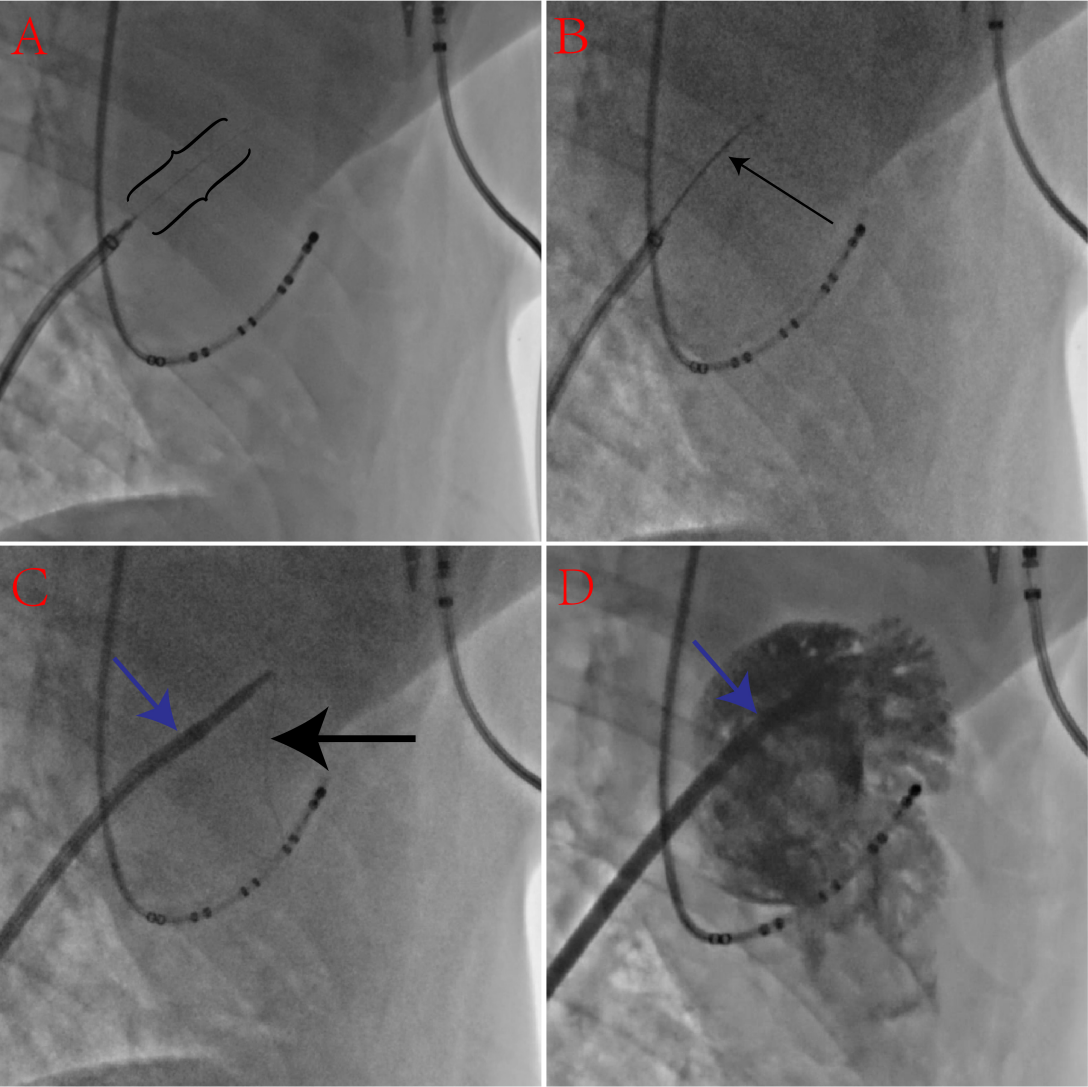
**A sequence of X-ray images showing transseptal puncture with a percutaneous transluminal coronary angioplasty (PTCA) guidewire and a microcatheter in pigs**. (A) The back end of the PTCA guidewire (black brackets) passes through the fossa ovalis (FO) into the left atrium (LA) through a microcatheter. (B) The microcatheter (thin black arrow) advances over the PTCA guidewire into the LA. (C) Advancement of the dilator and sheath (blue arrow) into the LA over the wire (thick black arrow). (D) Contrast injected into the LA.

The FO was punctured with the back end of a 0.014 inch Sion PTCA guidewire and a 2.3 Fr Cosair microcatheter. The punctures were successful within two attempts. As soon as the guidewire contacts the FO, the guidewire could puncture through the FO into the LA in less than 1 min (Fig. 1A). The microcatheter was then quickly advanced into the LA following the guidewire in several seconds (Fig. 1B). After the soft front end of the guidewire was exchanged into the LA, the guidewire was curled inside the LA without any concern for accidental puncture or tears. Finally, the sheath and dilator were introduced through the FO into the LA (Fig. 1C), and LA access was confirmed by contrast injection (Fig. 1D).

**Fig. 2.**
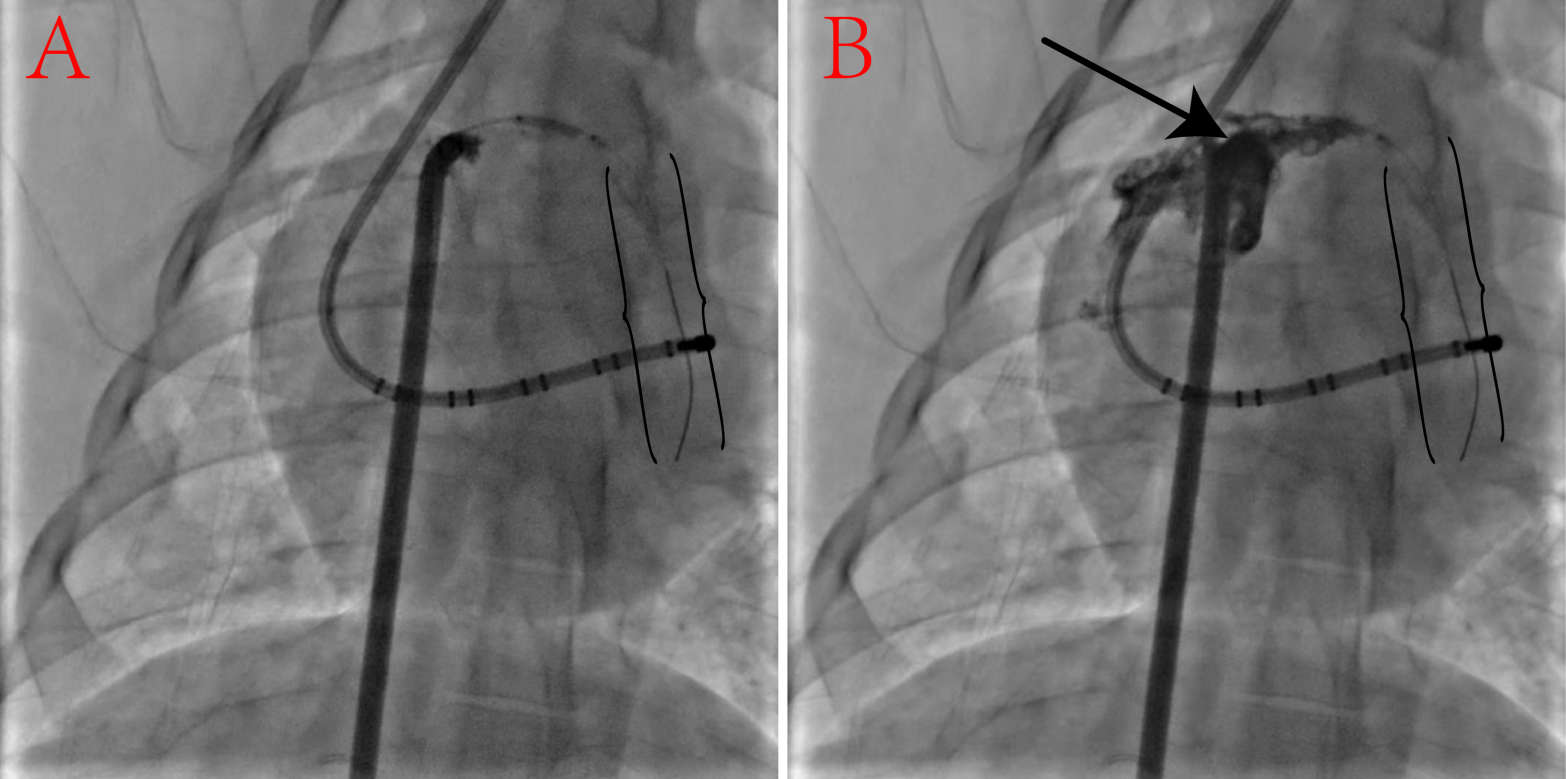
**Transvenous puncture of the left atrial appendage (LAA) to test the safety margin of this method**. (A) The LAA was intently punctured by the back end of PTCA guidewire (black brackets), and a 1.5 mm × 15 mm balloon was used to expand the puncture hole (12 atm × 15 min). (B) Balloon was dilating the puncture hole (black arrow) and contrast was injected to confirm the balloon position. PTCA, percutaneous transluminal coronary angioplasty.

The safety test showed the method had an acceptable safety margin. The LAA was intentionally punctured by the guidewire, followed by a balloon to expand the puncture hole. Subsequently, contrast was introduced through the sheath to verify the balloon’s placement (Fig. 2). After the balloon was removed, the animals were examined for 30 min, and an echocardiogram revealed no evidence of pericardial effusion.

### 3.2 Application in a Challenging Case of Human TSP

**Fig. 3.**
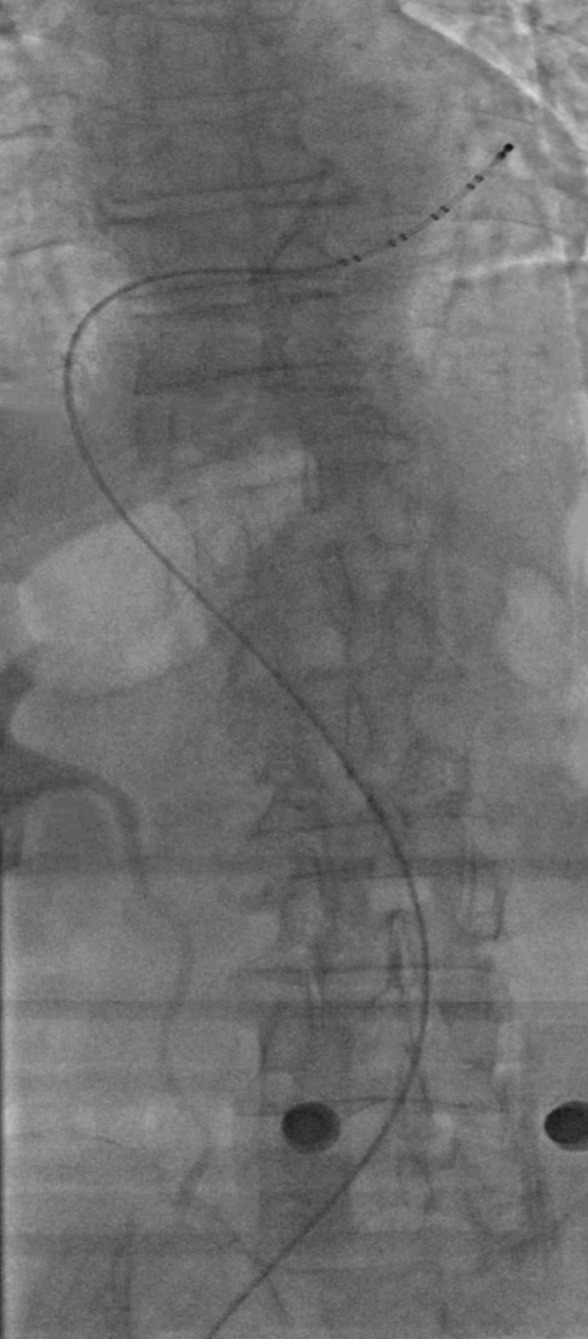
**Image of a very tortuous inferior vena cava**.

**Fig. 4.**
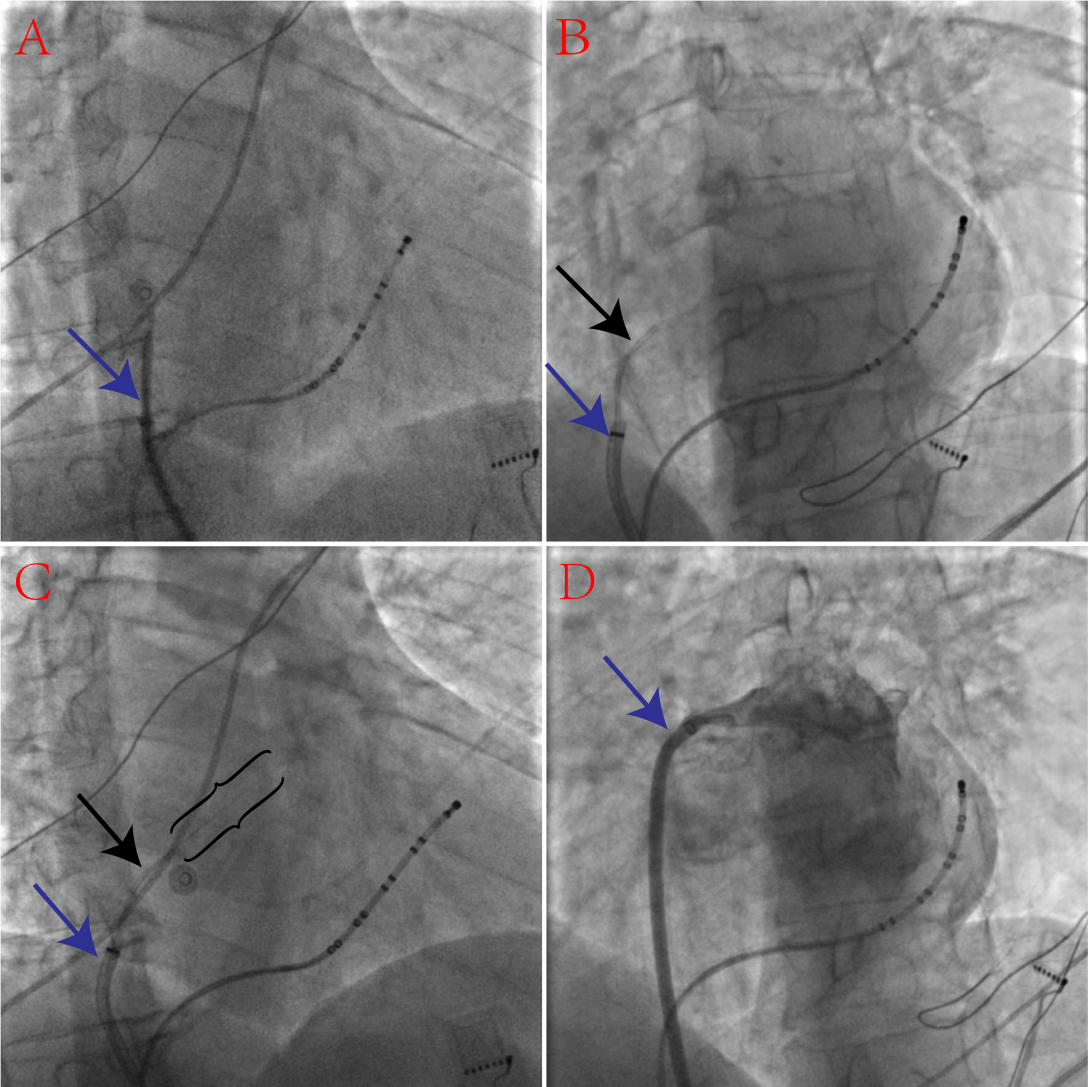
**A sequence of X-ray images showing transseptal puncture with a percutaneous transluminal coronary angioplasty (PTCA) guidewire and a microcatheter in a patient with tortuous inferior vena cava for atrial fibrillation ablation**. (A) The tips of the dilator and sheath (puncture needle within it) (blue arrow) cannot reach the fossa ovalis (FO) because of the vena cava is very tortuous. (B) The tip of a Tiger (TIG) angiographic catheter (black arrow) reaches the FO through the sheath (blue arrow). (C) The back end of a PTCA guidewire (black brackets) punctured through the FO. (D) The angiography of the left atrium (LA) and pulmonary vein.

During a procedure for pulmonary vein isolation (PVI) ablation in a male patient, resistance to the BRK needle advancemed into the dilator was detected. Fluoroscopy revealed extreme tortuosity of the inferior vena cava (Fig. 3) (**Supplementary video 1**), and the tip of the puncture needle could not reach the FO due to the inferior vena cava (Fig. 4A) (**Supplementary video 2**). After several unsuccessful attempts (including shaping the BRK needle tip and increasing the curvature) and with the consent of the patient’s family, a needle-free method was employed, using the back end of a PTCA guidewire and a microcatheter.

A 5 Fr TIG angiographic catheter was introduced through the sheath to engage the FO (Fig. 4B). The back end of a 0.014 inch Sion PTCA guidewire within the 2.3 Fr Cosair microcatheter was then advanced through the angiographic catheter to puncture the FO and enter the LA (Fig. 4C) (**Supplementary video 3**). The microcatheter entered the LA through the guidewire and access was confirmed with contrast injections. Next, the PTCA guidewire’s back end was exchanged with the soft front end. The TIG angiographic catheter entered the LA with the support of the guidewire and microcatheter. After the withdrawal of the guidewire and microcatheter was complete, a 0.032 inch “J” guidewire was advanced through the angiographic catheter into the LSPV to guide the dilator and sheath into the LA (**Supplementary video 4**). Finally, the sheath entered the LA, and LA angiography was performed (Fig. 4D) (**Supplementary video 5**); PVI was performed as usual. In this difficult TSP case, the time taken to perform the TSP procedure was 12 min (from the PTCA guidewire reaching the FO to the SL1 sheath entering the LA). No complications occurred during the procedure.

## 4. Discussion

The findings of this study demonstrate that TSP performed with a PTCA guidewire and microcatheter is feasible in specific conditions. Without a puncture needle, the back end of the PTCA guidewire can puncture the FO with the support of the sheath and catheter. Moreover, the subsequent passage of the sheath is smooth. The animal safety study demonstrated this method had an acceptable margin of safety.

### 4.1 Feasibility and Safety of the PTCA Guidewire for TSP

The blunt guidewire can penetrate through the FO owing to its small diameter and the support of the catheter. While the guidewire is flexible and blunt, the support of the catheter can greatly increase guidewire penetration. This method is often employed in percutaneous coronary procedures for chronic complete blockage. When the technique is used for TSP, support from one catheter in a sheath may be insufficient, and other catheters can be included to improve support. *In vitro*, a 5 Fr catheter can be introduced first into a 6 Fr guiding catheter, then into an 8 Fr guiding catheter, and finally, into an 8.5 Fr sheath for stronger support. In this study, another catheter was not used to improve support, because the pigs were young the FO was weak.

The guidewire is easier to manipulate than the BRK needle. It advances through the guiding catheter and can be controlled by the direction of the sheath and catheter, which is especially helpful in the case of inferior vena cava tortuosity. Previous studies have emphasized the risk of needle puncture in tortuous veins [10]. The BRK needle is bulky and requires a relatively long learning curve. The failure rate tends to be high for beginners. Moreover, in some cumbersome procedures it may be difficult to adjust direction, and the entire device must be removed in order to repeat the puncture, which may lead to the derailment of the puncture equipment.

In theory, the PTCA guidewire may be safer than the BRK needle for TSP. The BRK needle is rigid and sharp, and therefore, risks tearing the heart during TSP. Additionally, translational respiratory and the torsion of cardiac motion can further increase the risk of laceration [9]. Rather than from direct needle penetration, the majority of severe bleeding complications are caused by lacerations from the heart as it pulsates against the pointed tip of the needle [11]. These lacerations, resulting from sharp instruments, typically create neat incisions; the myocardial tissue is entirely cut through, and the contractions of the heart are unable to seal the wound. In fact, under the conditions of cardiac motion, these wounds can further enlarge. If an inadvertent puncture of adjacent structures is noted and handled in a timely manner, a catastrophe complication may be averted [4, 12, 13, 14]. Previous research has shown that in a pig model, the right ventricle can control bleeding from a puncture hole of 2.5 mm, and the right atrial appendage can manage bleeding from a puncture hole the size of a 4 Fr catheter [9, 15]. Our study showed that the LAA could control bleeding after the puncture site was expanded with a 1.5 mm balloon. Reasons why ordinary puncture wounds generally do not cause severe bleeding include: The myocardium’s inherent hemostatic ability: due to the complex three-dimensional network of myocardial fibers, this structure aids in spontaneous hemostasis when the myocardium suffers a puncture injury without extensive tissue laceration. Additionally, the circumferentially oriented muscle fibers on the epicardial surface can control bleeding through fiber contraction [16]. Moreover, the smaller the puncture hole, the less harmful the procedure; the diameter of the guidewire is much smaller than that of the puncture needle. On the other hand, the PTCA guidewire is flexible and blunt and can track the motion of a beating heart, which greatly reduces the risk of laceration. The penetrability of the guidewire is greatly decreased after it enters the LA because it loses catheter support; therefore, the probability of an accidental puncture and tear is low. Furthermore, the back end of the PTCA guidewire will be exchanged with the softer front end after confirming establishment of access to further ensure the safety of the procedure [17, 18]. The advancement of the microcatheter also serves to envelop the end of the guidewire, thereby reducing the risk of accidental punctures. It’s worth noting that we used a soft Sion wire. Using stiffer tip wires could increase the risk of chamber perforation and should only be attempted in cases where a soft wire cannot penetrate the FO. Even then, such attempts should be made with utmost caution.

### 4.2 Other Methods to Accomplish TSP

Other technologies for TSP have been described; however, not all medical centers are equipped with suitable equipment and technical personnel. The PTCA guidewire and microcatheter are relatively inexpensive and easily accessible, almost every cardiac catheterization lab is equipped with one. The SafeSept guidewire is a notable improvement from the original TSP in that it requires less force and is safer than traditional puncture needles [19, 20]. There are two models of the SafeSept guidewire available: the first requires alignment with the puncture needle and the second is a tapered wire, which is limited by not allowing contrast to be injected through the wire to determine the location, it can confirm the puncture site by reaching into the pulmonary vein, but sometimes it can not reach into the pulmonary vein, which may necessitate the use of ICE to confirm the correct puncture site. Our method can confirm the puncture site under fluoroscopy alone by injecting contrast through the microcatheter or positioning the guidewire into the pulmonary vein. Knadler *et al*. [21] reported a case involving a major complication with the SafeSept guidewire, which crossed from the aorta into the coronary artery system. Other technologies also use RF energy to facilitate transseptal passage [7, 22, 23, 24]. Justino *et al*. [25] and Benson *et al*. [26] used a dedicated RF wire for atrial septum perforation. Its particular advantages are evident in small children, patients with thick atrial septums, and in cases involving tortuous or a defective inferior vena cava, where traditional needle techniques may be less effective or even unfeasible.

### 4.3 Clinical Implications

TSP is a basic technique for cardiac interventional procedures, such as left-sided arrhythmia ablation, left atrial appendage closure, percutaneous left ventricular assisted device insertion, and mitral valve procedures. An increasing number of interventional cardiac procedures will be performed at less experienced centers and many new inexperienced physicians will need to complete TSP training. However, some centers do not use TEE or ICE to guide procedures, especially in some developing countries; therefore, improving technologies is important to ensure patient safety and increase the efficiency of the clinical procedures. This study demonstrates several findings. First, a PTCA guidewire and a microcatheter may be used to perform TSP without a puncture needle. Second, as a supplementary method, it can be considered for use in cases such as an extremely tortuous inferior vena cava, or when needle fails. Third, this method has an acceptable safety margin.

### 4.4 Limitations

This study has several limitations. First, the method was tested in a small sample size of pigs and in only one patient. A larger patient cohort study is required to evaluate the validity and safety of the method. Our objective in this study is to describe this method, with the aim of helping patients with difficult TSP, especially those presenting with extreme tortuosity of the inferior vena cava. Second, because the pigs were 3 months old, their FO were weak, this reduced the difficulty of puncture. In certain conditions such as mitral stenosis or chronic LA overload, FO can be very thick and may not be penetrable by a PTCA guidewire. Thus, the feasibility of this method in the case of thick FO warrants further investigation. Third, some steps are relatively redundant and need to be further simplified. Fourth, the PTCA guidewire and microcatheter are off label uses and not specifically designed for TSP; therefore, some improvements are required such as adjusting the X-ray transmittance and shape of the guidewire.

## 5. Conclusions

TSP performed with the back end of a PTCA guidewire and a microcatheter, without a BRK needle, may be feasible and may have an acceptable safety margin. Although further evaluation is required, this approach has the potential to be a supplementary method in special conditions, such as in cases presenting with an extremely tortuous inferior vena cava.

## Data Availability

The original contributions presented in the study are included in the article and/or in the Supplementary video material. Further inquiries can be directed to the corresponding author/s.

## Abbreviations

BRK, Brockenbrough; CS, Coronary sinus; FO, Fossa ovalis; ICE, Intracardiac echocardiography; LA, Left atrium; LAA, Left atrial appendage; LAO, Left anterior oblique; LSPV, Left Superior pulmonary vein; PTCA, Percutaneous transluminal coronary angioplasty; PVI, Pulmonary vein isolation; RF, Radiofrequency; RV, Right ventricle; TEE, Transesophageal echocardiography; TSP, Transseptal puncture.

## Supplementary Material

Supplementary material associated with this article can be found, in the online version, at https://doi.org/10.31083/j.rcm2505170.
