# Supplementary material for: Needle-free, Novel Fossa Ovalis Puncture with Percutaneous Transluminal Coronary Angioplasty Guidewire and Microcatheter in Pigs and a Human with an Extremely Tortuous Inferior Vena Cava
Source: Rev Cardiovasc Med. 2024 May 14;25(5):170. doi: 10.31083/j.rcm2505170 (PMC11267186; doi:10.31083/j.rcm2505170)
Supplement: Supplementary file 1 [file 2153-8174-25-5-170-s1.zip › 2153-8174-25-5-170-s1/Supplementary Material.docx]

Supplementary Material

Supplementary video 1: The catheter is in a very tortuous inferior vena cava.

Supplementary video 2: The fossa ovalis (FO) of the patient cannot be engaged by the tip of the dilator and sheath with the puncture needle embedded.

Supplementary video 3: The back end of a percutaneous transluminal coronary angioplasty (PTCA) guidewire within the microcatheter advances through the 5 Fr TIG angiographic catheter to puncture the fossa ovalis (FO) and enter the left atrium (LA) in the case of difficult transseptal puncture (TSP).

Supplementary video 4: A “J” guidewire advances to the left superior pulmonary vein (LSPV) through the 5 Fr TIG angiographic catheter after it passes the fossa ovalis (FO).

Supplementary video 5: The angiography of left atrium (LA) and pulmonary vein of the patient.
